# *Monodopsis subterranea* is a source of α‐tocomonoenol, and its concentration, in contrast to α‐tocopherol, is not affected by nitrogen depletion

**DOI:** 10.1002/fsn3.3880

**Published:** 2023-12-20

**Authors:** Alexander Montoya‐Arroyo, Alejandra Muñoz‐González, Katja Lehnert, Konstantin Frick, Ulrike Schmid‐Staiger, Walter Vetter, Jan Frank

**Affiliations:** ^1^ Institute of Nutritional Sciences (140b) University of Hohenheim Stuttgart Germany; ^2^ School of Food Technology University of Costa Rica San Pedro Costa Rica; ^3^ Institute of Food Chemistry (170b) University of Hohenheim Stuttgart Germany; ^4^ Institute of Interfacial Process Engineering and Plasma Technology University of Stuttgart Stuttgart Germany; ^5^ Innovation Field Functional Ingredients Fraunhofer Institute for Interfacial Engineering and Biotechnology IGB Stuttgart Germany

**Keywords:** marine‐derived tocopherol, microalgae, tocotrienol, vitamin E

## Abstract

α‐Tomonoenols (αT1) are tocochromanols structurally related to tocopherols (T) and tocotrienols (T3), the bioactive members of the vitamin E family. However, limited evidence exists regarding the sources and biosynthesis of tocomonoenols. Nitrogen depletion increases the content of α‐tocopherol (αT), the main vitamin E congener, in microalgae, but little is known regarding its effect on other tocochromanols, such as tocomonoenols and tocotrienols. We therefore quantified the concentrations of T, T1, and T3, in freeze‐dried biomass from nitrogen‐sufficient, and nitrogen‐depleted *Monodopsis subterranea* (Eustigmatophyceae). The identities of isomers of αT1 were confirmed by LC–MS and GC–MS. αT was the predominant tocochromanol (82% of total tocochromanols). αT1 was present in higher quantities than the sum of all T3 (6% vs. 1% of total tocochromanols). 11′‐αT1 was the main αT1 isomer. Nitrogen depletion increased αT, but not αT1 or T3 in *M. subterranea*. In conclusion, nitrogen depletion increased the content of αT, the biologically most active form of vitamin E, in *M. subterranea* without affecting αT1 and T3 and could potentially be used as a strategy to enhance its nutritional value but not to increase αT1 content, indicating that αT1 accumulation is independent of that of αT in microalgae.

## Graphical abstract

α‐Tocomonoenols are tocochromanols with high structural similarity to α‐tocopherol, the main vitamin E congener. In this paper, we demonstrate that the cultured microalgae *Monodopsis subterranea* is a source of α‐tocomonoenols, particulary 11′‐α‐tocomonoenol, and that the accumulation of this congener is independent of that of α‐tocopherol, as shown by cultivation under nitrogen depletion.

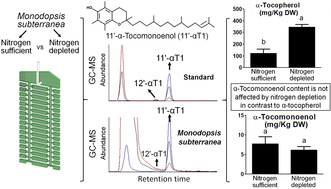

## 1 INTRODUCTION

**FIGURE 1:**
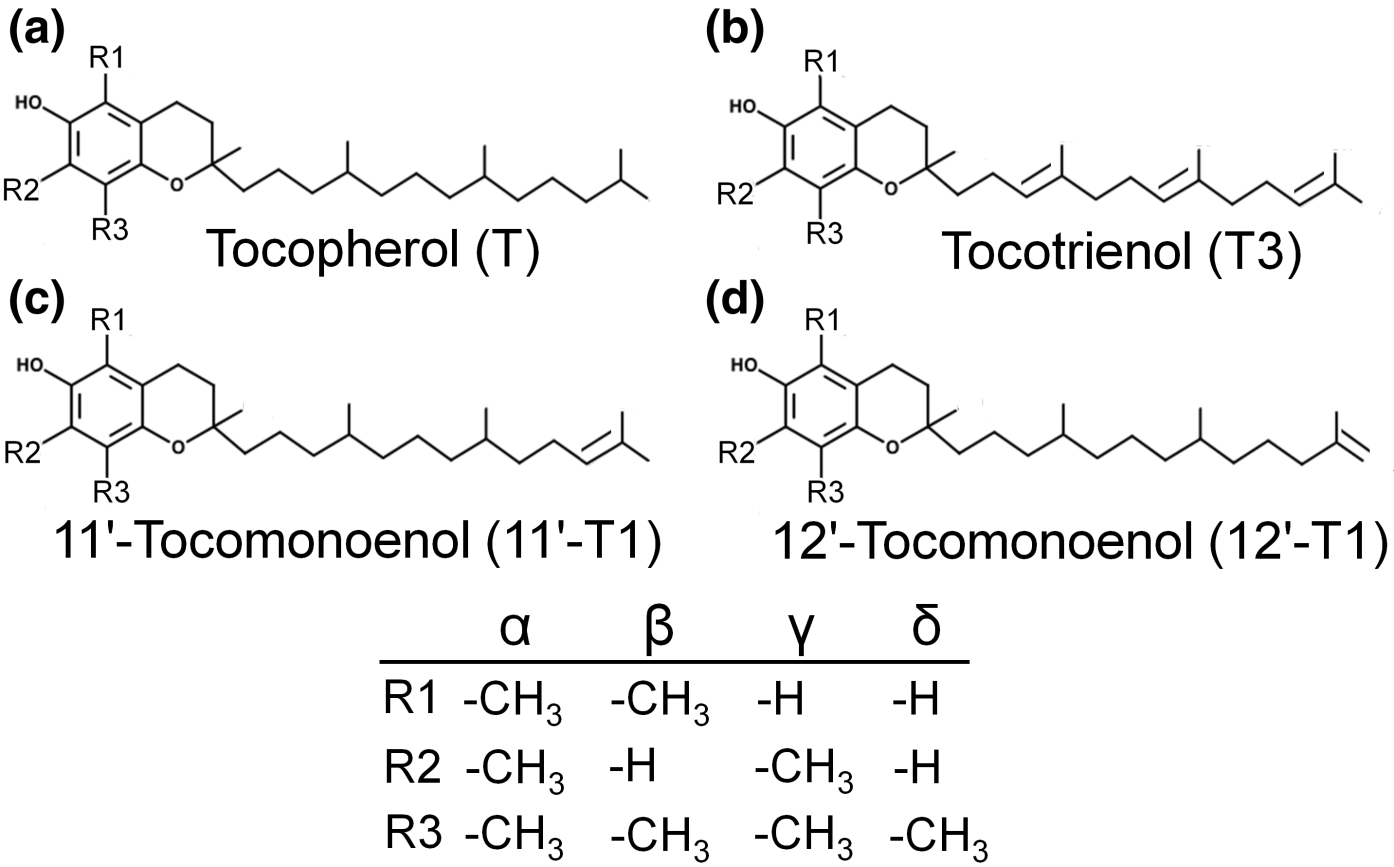
Chemical structures of tocopherols (T) (a); tocotrienols (T3) (b); 11′‐tocomonoenols (11′‐T1) (c); and 12′‐tocomonoenols (12′‐T1) (d).

Tocopherols (T), tocomonoenols (T1), and tocotrienols (T3) are structurally related liposoluble compounds collectively referred to as tocochromanols. They all share a chemical structure comprised of a chromanol ring and a 16‐carbon sidechain. The sidechain of T is saturated, whereas T1 and T3 carry a single or three double bonds, respectively, in theirs (Figure 1). Based on the number and positions of methyl groups substituted at the chromanol ring, α‐, β‐, γ‐, and δ‐congeners of the respective tocochromanols are distinguished (Birringer et al., 2018).

αT is generally considered as the tocochromanol with the highest (EFSA, 2015), if not the only tocochromanol with vitamin E activity, despite different reported biological activities for T3 and other non‐αT congeners (Azzi, 2019). Structural features, such as the methylation pattern of the chromanol ring and degree of saturation of the sidechain, impact bioavailability (Sontag & Parker, 2007), and protein recognition (Hosomi et al., 1997), which are among the relevant factors involved in tocochromanol bioactivity. T1 are quantitatively minor tocochromanols present in foods (Butinar et al., 2011; Matsumoto et al., 1995; Yamamoto et al., 1999), with αT1 presenting great structural similarity to αT (Birringer et al., 2018). Two different αT1 isomers only differing in the position of the double bond in the sidechain have been discovered so far, namely 11′‐αT1 (Matsumoto et al., 1995) and 12′‐αT1 (Figure 1) (Yamamoto et al., 1999).

11′‐αT1 has been found in vegetable oils (Butinar et al., 2011; Hammann et al., 2015; Irías‐Mata et al., 2017), processed foods containing vegetable oils (Mignogna et al., 2015), the deodorized scum of tuna fish oil (Gotoh et al., 2009), and in cultured microalgae of the genera *Nannochloropsis* (Eustigmatophyceae), *Tetraselmis*, and *Chlorella* (Chlorophyceae) (Montoya‐Arroyo, Lehnert, Lux, et al., 2022; Montoya‐Arroyo, Lehnert, Muñoz‐González, et al., 2022). 12′‐αT1 was initially termed marine‐derived tocopherol (MDT) and has been reported in fish, fish eggs, krill, phytoplankton (Dunlap et al., 2002; Yamamoto et al., 1999, 2001), and in cultured microalgae (Montoya‐Arroyo, Lehnert, Lux, et al., 2022; Montoya‐Arroyo, Lehnert, Muñoz‐González, et al., 2022).

Even though the biological importance of tocomonoenols has yet to be determined, it has been demonstrated that 11′‐αT1 is taken up into and metabolized by hepatocytes similar to αT (Montoya‐Arroyo et al., 2021). Because the metabolism of 11′‐αT1 mirrors that of the main vitamin E congener αT, it may also share some of its biological functions. In addition, some activities, such as the modulation of adipocyte differentiation and activation of immune cells, have already been reported for 12′‐αT1 (Beppu et al., 2020). Nevertheless, limited knowledge regarding the determinants of the biosynthesis of αT1 exists. Due to the increasing interest in the bioactive potential of T1 (Azzi, 2019), a better understanding of sources and biosynthetic determinants requires further attention.

*Monodopsis subterranea* (Eustigmatophyceae) is a freshwater microalga of industrial interest due to its high content of the omega‐3 fatty acid eicosapentaenoic acid (Arora & Mishra, 2019), since the main dietary sources of omega‐3 fatty acids, namely fish and marine products, are threatened by overfishing (Tocher et al., 2019). The presence of αT1 isomers reported in microalgae from Eustigmatophyceae (Montoya‐Arroyo, Lehnert, Lux, et al., 2022; Montoya‐Arroyo, Lehnert, Muñoz‐González, et al., 2022) suggests that these congeners might also be present in other related microalgae such as *M. subterranea*. Nevertheless, limited knowledge exists regarding the tocochromanol content of *M. subterranea* and whether or not it contains T1. Nitrogen depletion is a culture strategy that increases total lipids (Janssen et al., 2019; Meng et al., 2015; Ördög et al., 2016), eicosapentanoic acid (EPA) content in triacylglycerols (Janssen et al., 2019; Meng et al., 2015), and αT concentrations in microalgae (Goiris et al., 2015; Mudimu et al., 2017). However, it is currently unknown if nitrogen depletion also affects the concentration in microalgae of other tocochromanols, such as T1.

The aim of this study was, therefore, to investigate the potential presence of αT1 in *M. subterranea* and the impact of nitrogen depletion on its tocochromanol content and profile. We thus hypothesized that *M. subterranea* contains αT1 and that its tocochromanol content will increase under nitrogen depletion conditions as it occurs with αT.

## 2 MATERIALS AND METHODS

### 2.1 Standards

Commercial standards of RRR‐α‐tocopherol, RRR‐β‐tocopherol, RRR‐δ‐tocopherol, RRR‐γ‐tocopherol (≥95%; Sigma‐Aldrich, Taufkirchen, Germany), α‐tocotrienol, β‐tocotrienol, δ‐tocotrienol, γ‐tocotrienol (≥97%; Sigma‐Aldrich, Taufkirchen, Germany), and isolated 11′‐αT1 (≥97%) extracted from a palm oil vitamin E extract (Müller et al., 2018) were used for tocochromanol quantification.

### 2.2 Microalgae strain

*Monodopsis subterranea* (Eustigmatophyceae) strain SAG 848‐1 was obtained from the Department of Experimental Phycology and Culture Collection of Algae at the Georg‐August University in Göttingen, Germany (EPSAG, 2023) and cultured at the Fraunhofer Institute for Interfacial Engineering and Biotechnology (Stuttgart, Germany), as described in the following sections.

### 2.3 Culture conditions

Cultivation was performed under nitrogen‐sufficient and nitrogen‐depleted conditions, as previously described. *M. subterranea* was cultivated in freshwater using modified OHM medium (Fábregas et al., 2000) with 493 mg/L MgSO_4_·7 H_2_O; 222 mg/L CaCl_2_·2 H_2_O; 5.2 mg/L Fe (III)citrate·5 H_2_O; 0.02 mg/L CoCl_2_·6 H_2_O; 0.024 mg/L CuSO_4_·5 H_2_O; 1.98 mg/L MnCl_2_·4 H_2_O; and 0.24 mg/L Na_2_MoO_3_·2 H_2_O. Phosphorus was added separately from a phosphate stock solution (50 g/L). The phosphate content in the media ranged between 20 and 200 mg/L during the experiments. Nitrogen was added separately using an ammonium stock solution (35 g/L). During the experiments, the ammonium concentration ranged between 30 and 300 mg/L in a nitrogen‐sufficient culture. In nitrogen‐depleted conditions, the addition of ammonium was stopped.

*Monodopsis subterranea* was cultivated in flat panel airlift photobioreactors (Subitec GmbH, Germany) with a volume of 6 L. The cultivation conditions were monitored and controlled via an automated control unit (Siemens SPS, Germany). During the cultivation, the culture temperature ranged between 20 and 20.5°C, and the pH was kept between 7.1 and 7.5 by adding CO_2_ (1–20 L/h) to the gas flow (0.5–0.55 vvm). The amount of light per gram of biomass (specific light availability) was kept steady at 5 μmol_photons_/(g_DW_·s) by adjusting the light intensity daily, as described by Holdmann et al. (2018). Light intensity on the reactor surface was therefore increased with increasing biomass concentration of the culture and ranged from 140 to 430 μmol_photons_/(m^2^·s) during cultivation of the pre‐cultures and from 140 to 1050 μmol_photons_/(m^2^·s) during cultivation of the experimental cultures.

#### 2.3.1 Pre‐cultures

To avoid any adaptation processes during the experiments, microalgae cultures were inoculated from an established pre‐culture, cultivated in a flat‐panel airlift photobioreactor as described above (Section 2.3). Through regular dilution, the biomass concentration was kept between 1 and 3 g/L. The pre‐culture was grown under the described conditions for at least 14 days before being used as an inoculum for further experiments.

#### 2.3.2 Nitrogen‐sufficient samples

*Monodopsis subterranea* was inoculated from a pre‐culture and cultivated for 4 days under the cultivation conditions described above (Section 2.3). After 4 days, the culture from this first batch was diluted to 1 g/L, and the experiment was restarted subsequently until three batches in total were obtained. Biomass from three independent batches was collected and pre‐processed independently.

#### 2.3.3 Nitrogen‐depleted cultures

Three *M. subterranea* cultures were inoculated with the biomass from the third batch of nitrogen‐sufficient experiments (see Section 2.3.2). The cultivation conditions were similar to the nitrogen‐sufficient conditions, but the addition of ammonium was stopped in these experiments. The cultures were harvested after the biomass concentration stopped increasing (17 days). For analyses, biomass from three independent bioreactors was collected and pre‐processed independently as independent batches.

### 2.4 Sample pre‐treatment

Biomass was harvested from the culture medium by centrifugation. The supernatant was discarded and the pellet freeze‐dried (Christ Alpha 1‐2 LD freeze drier, Osterode am Harz, Germany) (Gille et al., 2019). Freeze‐dried samples were stored in polyethylene bags, vacuum‐sealed, and protected from light at −20°C. Prior to analyses, samples were ground using an analytical laboratory mill (IKA A11; IKA‐Werke GmbH & Co, Staufen, Germany) and stored at −80°C, protected from light and moisture.

### 2.5 Tocochromanol extraction

Tocochromanols were extracted as described previously (Montoya‐Arroyo, Lehnert, Lux, et al., 2022). Freeze‐dried samples (100 mg) were incubated with continuous shaking (30 min at 70°C) in the presence of ascorbic acid 1% (m/v) in ethanol and saturated aqueous KOH. After cooling on ice, samples were neutralized with glacial acetic acid, mixed with butylhydroxytoluene as an antioxidant, and tocochromanols were cold‐extracted with 2 mL of *n*‐hexane. Samples were mixed, inverted repeatedly during 1 min, and centrifuged (280 *g*, 5 min, 4°C, microcentrifuge Heraeus Fresco 17; Thermo Fischer Scientific, Waltham MA, USA) for phase separation. The organic upper layer was recovered, and extraction was repeated 3 additional times. Pooled organic phase was evaporated to dryness in a vacuum centrifuge (RVC 2‐25 CD Plus, Martin Christ Gefriertrocknungsanlagen), and samples were re‐suspended in 100 μL ethanol, mixed, transferred to an Eppendorf tube, cooled on ice, and centrifuged (17,000 *g*, 10 min, 4°C, microcentrifuge Heraeus Fresco 17; Thermo Fischer Scientific, Waltham MA, USA) to obtain clear ethanolic suspensions containing tocochromanols. Samples were extracted in triplicate per batch and triplicate batches per treatment.

### 2.6 Tocochromanol quantification by high‐performance liquid chromatography with fluorescence detection

Clear ethanolic suspensions containing tocochromanols (20 μL) (see Section 2.5) were automatically injected into a Jasco HPLC system (JASCO Deutschland GmbH Labor‐ und Datentechnik, Pfungstadt, Germany) equipped with a fluorescence detector (excitation/emission wavelengths = 296/325 nm) and a Kinetex PFP column (100 × 4.6 mm i.d., 2.6 μm particle size; Phenomenex, California, USA) at 40°C. A mixture of methanol/water (76/24 [v/v]) with a flow rate of 1.2 mL/min was used as the mobile phase in a 90 min run. Commercial standards of T and T3 and an isolated standard of 11′‐αT1 were used for identification and quantification. Samples were analyzed in triplicate per batch and triplicate batches per treatment.

### 2.7 Tocochromanol identification by liquid chromatography–atmospheric pressure‐chemical ionization mass spectrometry

LC–MS identification of tocochromanols was done as previously described (Montoya‐Arroyo, Lehnert, Lux, et al., 2022). Ethanolic suspensions containing tocochromanols (Section 2.5) were diluted with ethanol and automatically injected (5 μL) into a 1290 series HPLC system (Agilent, California, USA) equipped with a Kinetex PFP column (100 × 4.6 mm i.d., 2.6 μm particle size; Phenomenex, Aschaffenburg, Germany) maintained at 40°C and a photodiode array detector (wavelength range: 190–600 nm). The HPLC system was connected to a Q Exactive Plus – Orbitrap mass spectrometer equipped with an atmospheric pressure chemical ionization source in positive mode with a scan range of *m*/*z* 100–1000 in full scan mode and *m*/*z* 50–1000 for MS^2^ determinations. A binary gradient elution system (Eluent A: methanol/water [80/20, v/v]; Eluent B: methanol/water [97/3, v/v]) with a total flow rate of 0.6 mL/min was used as the mobile phase. The proportion of eluent B increased from 0% to 100% within 20 min, was held at 100% for 5 min, backflushed to 0% within 2 min, and was held at 0% until the end of the run time (30 min). Commercial standards of T and T3 and isolated standard of 11′‐αT1 were used for tocochromanol identification.

### 2.8 11′‐αT1 identification by gas chromatography–mass spectrometry

Samples were prepared as shown previously (Montoya‐Arroyo, Lehnert, Lux, et al., 2022). In brief, freeze‐dried microalgae biomass (200 mg) was cold‐extracted with *n*‐hexane, the solvent was changed to 2 mL of ethanol supplemented with pyrogallol (60 g/L; Carl Roth, Karlsruhe, Germany). After carefully performed washing steps, the remaining sample with 11′‐α‐T1 was silylated according to Hammann et al. (2016). The liquid phase was evaporated, and the dried residue was taken up with exactly 100 μL of *n*‐hexane supplemented with the internal standard 5α‐cholestane (1.3 μg/mL; Sigma‐Adrich, Taufkirchen, Germany). Subsequent gas chromatography–mass spectrometry (GC–MS) analysis and identification of 11′‐α‐T1 were performed according to Müller et al. (2018). Briefly, 1 μL sample solution was automatically injected into a 6890/5973 system (splitless injections at 250°C) (Hewlett‐Packard/Agilent, Waldbronn, Germany). An OPTIMA 5 MS capillary column (30 m length, 0.25 mm internal diameter, 0.25 μm film thickness; Macherey‐Nagel, Düren, Germany) was used with the GC oven program described by Hammann et al. (2016). In GC–MS full scan mode (*m*/*z* 50–500), each sample was analyzed in triplicate. Isolated 11′‐αT1 containing traces of 12′‐αT1 was used for αT1 identification as previously described (Müller et al., 2018).

### 2.9 Chlorophyll quantification

One‐hundred milligrams of freeze‐dried microalgae were transferred to a glass tube, mixed gently with 2 mL of HPLC‐grade ethanol, sonicated for 10 min in an ultrasonic bath, and centrifuged at 280 *g* for 7 min at 4°C. Then, 1 mL of the solvent was transferred to a volumetric flask covered with aluminum foil and kept on ice. Ethanolic extraction was repeated 5 additional times by adding 2 mL of ethanol, mixing, centrifuging, and recovering 2 mL of supernatant each time. All supernatants (11 mL) were pooled together and diluted in a 25 mL volumetric flask with ethanol. After agitation, 1 mL of the final solution was transferred to a glass cuvette, and the chlorophyll content was determined spectrophotometrically (UviLine 9400; SI Analytics GmbH, Mainz, Germany) by simultaneous reading of the absorbances at 632, 649, 665, and 696 nm using HPLC‐grade ethanol (Carl Roth, Karlsruhe, Germany) as the blank solution. For quantification, the equations proposed by Ritchie (2008) for the determination of chlorophyll in an ethanolic solution (Ritchie, 2008) were used. Samples were analyzed in triplicate per batch and triplicate batches per treatment.

### 2.10 Statistical analysis

Data are reported as the arithmetic mean ± standard deviation. Significant differences between arithmetic means were calculated using an independent sample *t*‐test with a significance level of *α* = .05 and a degree of freedom of df = 4. The mean values and standard deviations for each treatment were calculated using the three corresponding mean values from independent batches.

## 3 RESULTS AND DISCUSSION

*Monodopsis subterranea* contained all tocopherol (T) and tocotrienol (T3) congeners. αT1 was detected (Table 1, Figure S1) in both nitrogen‐sufficient and nitrogen‐depleted *M. subterranea*. In nitrogen‐sufficient biomass, the tocochromanol profile was dominated by T (93.5 ± 2.6% of total tocochromanols), with αT as the most abundant congener (80.9 ± 3.45% of total tocochromanols). αT1 corresponded to 5.5% of total tocochromanols, while total T3 reached only 1.00 ± 0.35% of total tocochromanols. αT3 (0.63 ± 0.26% of total tocochromanol content) was the main T3 congener (Table 1).

**TABLE 1:** Tocochromanol concentrations in nitrogen‐sufficient and nitrogen‐depleted *Monodopsis subterranea* obtained using HPLC‐FLD.

Congener	Concentration (mg/kg DW)		*p*‐Value
	Nitrogen‐sufficient	Nitrogen‐depleted	
α‐Tocopherol	122 ± 35.9	344 ± 24.7	.001
β‐Tocopherol	1.41 ± 0.24	2.18 ± 0.16	.010
γ‐Tocopherol	15.0 ± 4.74	51.8 ± 4.32	.001
δ‐Tocopherol	1.73 ± 0.37	4.34 ± 0.44	.001
α‐Tocomonoenol	7.64 ± 1.90	6.11 ± 0.97	.282
α‐Tocotrienol	0.88 ± 0.17	0.46 ± 0.01	.014
β‐Tocotrienol	0.09 ± 0.01	0.51 ± 0.46	.254
γ‐Tocotrienol	0.42 ± 0.03	0.27 ± 0.06	.017
δ‐Tocotrienol	nd	0.07 ± 0.08	.208
Total tocopherols	140 ± 40.4	402 ± 25.9	.001
Total tocotrienols	1.38 ± 0.19	1.31 ± 0.60	.846
Total tocochromanols	149 ± 38.7	410 ± 27.4	.001

*Note*: Values represent arithmetic means ± standard deviation of triplicate batches (*n* = 3 per batch). Significant differences between culture conditions were assessed by an independent sample *t*‐test (*α* = .05, degree of freedom = 4).

Abbreviation: nd, not detected.

**FIGURE 2:**
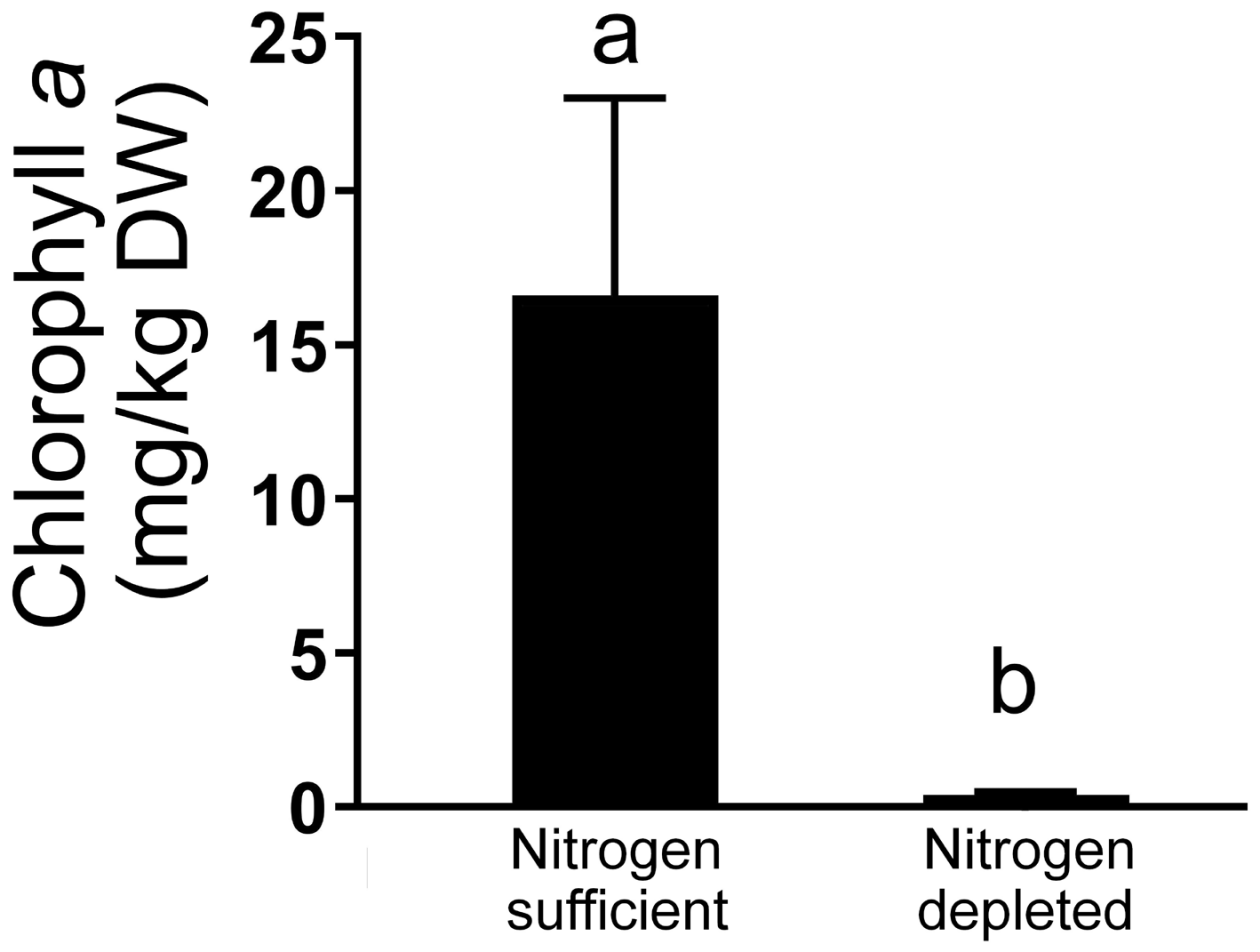
Effect of nitrogen depletion on the content of chlorophyll *a* in *Monodopsis subterranea*. Values represent the arithmetic mean ± standard deviation of triplicate batches (*n* = 3 per batch). Bars not sharing the same letter are significantly different based on a *t*‐test (*α* = .05, degree of freedom = 4).

After nitrogen depletion, the total tocochromanols and total T content of *M. subterranea* increased 2.7 and 2.8 times, respectively, mostly due to an increase in αT. The contents of γT and δT increased significantly by 3.5 and 2.5 times, respectively, without significant effects on βT. The observed increase in αT is in agreement with previous reports in other nitrogen‐depleted microalgae (Goiris et al., 2015; Mudimu et al., 2017; Singh et al., 2020). The 2.8‐fold increase in αT in nitrogen‐depleted *M. subterranea* is among the average changes previously reported for microalgae, ranging from 1.3 to 8.6 fold for different species (Mudimu et al., 2017). Tocopherol content also doubled in the leaves of the land plant *Arabidopsis thaliana* under N‐limitation (Gaude et al., 2007). Under nitrogen depletion, the chlorophyll content in *M. subterranea* decreased more than 40 times (Figure 2), which is also in agreement with previous reports in microalgae (Ferreira et al., 2016; Song et al., 2016) and is consistent with the reduction of chlorophyll content in leaves of nitrogen‐depleted *A. thaliana* (Gaude et al., 2007; Zhang et al., 2020).

**FIGURE 3:**
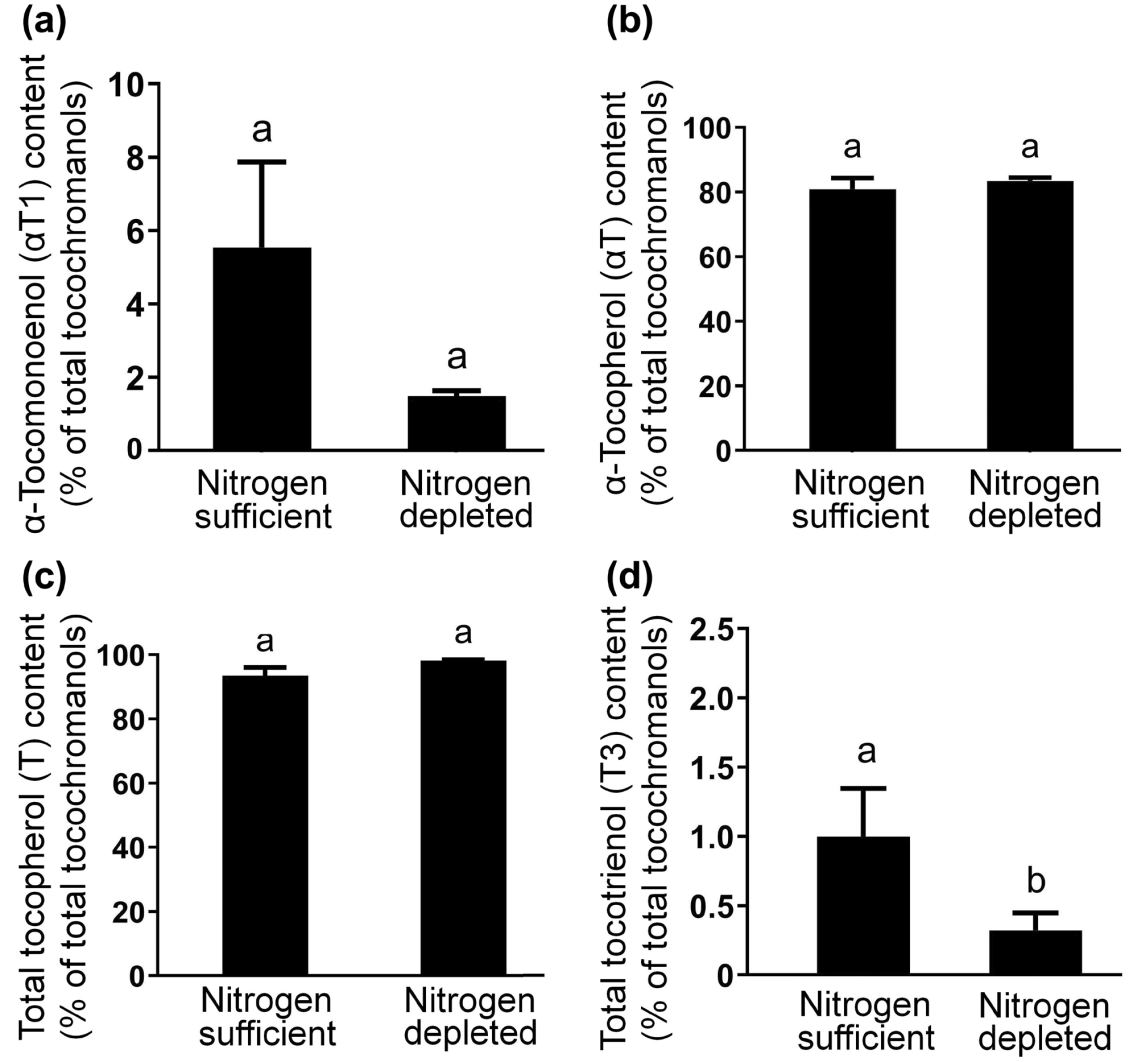
Effect of nitrogen depletion on the relative content (% of total tocochromanols) of α‐tocomonoenol (αT1) (a), α‐tocopherol (αT) (b), total tocopherols (T) (c), and total tocotrienols (T3) (d) in *Monodopsis subterranea*. Values represent the arithmetic mean ± standard deviation of triplicate batches (*n* = 3 per batch). Bars not sharing the same letter are significantly different based on a *t*‐test (*α* = .05, degree of freedom = 4). Content was determined using HPLC‐FLD.

No significant differences were observed in the absolute (*p* = .282; Table 1) or relative (*p* = .094) contents of αT1 (Figure 3A). The relative amounts of αT (Figure 3B) and total T (Figure 3C), as percentages of total tocochromanols, were not significantly affected by nitrogen depletion. Total T3 content remained similar in absolute terms (*p* = 0.846; Table 1) but decreased in relative terms (*p* = .033; Figure 3D). Under nitrogen depletion, βT3 was the predominant T3 in contrast to αT3 in nitrogen‐sufficient biomass (Table 1).

αT1 and T3 (Tables S1 and S2) in *M. subterranea* were confirmed using LC‐APCI‐MS by comparison with corresponding authentic standards of T3 and isolated 11′‐αT1. Signals of T3 in LC‐APCI‐MS were weak for effective ionization, and further optimization of extraction conditions might be required for unequivocal LC–MS^2^ confirmation of the quantitatively minor T3 observed in the tocochromanol profile after HPLC analysis using authentic commercial standards. Current data on standards and T3 identification by LC–MS are available in Appendix S1.

**FIGURE 4:**
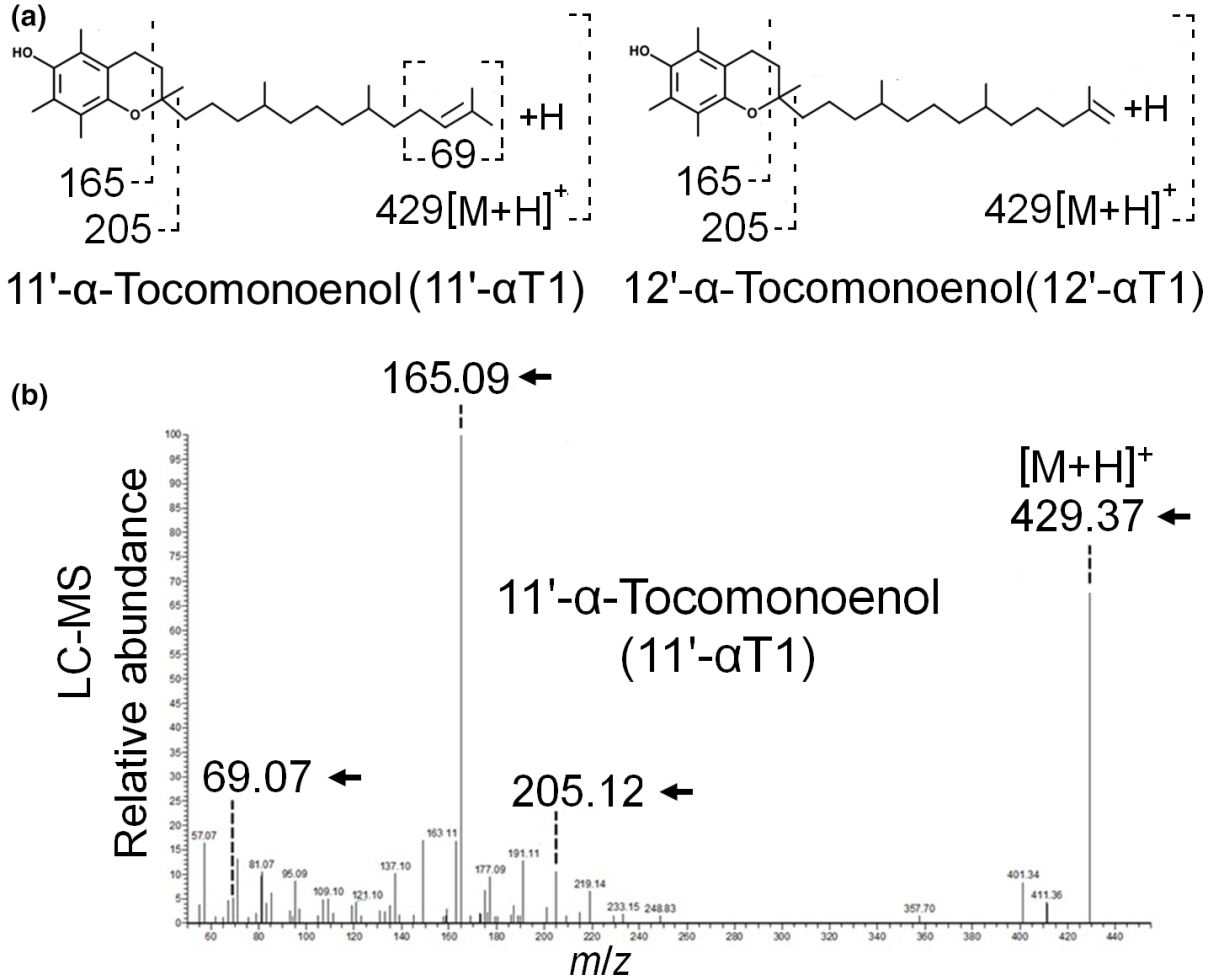
Chemical structures and expected fragmentation patterns of 11′‐α‐tocomonoenol (11′‐αT1) and 12′‐α‐tocomonoenol (12′‐αT1) (a). Representative MS^2^ spectra for the identification of αT1 in *Monodopsis subterranea* by LC‐APCI‐MS (b).

Figure 4 shows the characteristic fragmentation pattern expected for both 11′‐αT1 and 12′‐αT1 (Montoya‐Arroyo, Lehnert, Lux, et al., 2022) (Figure 4a) and a representative MS^*n*^ spectrum obtained from *M. subterranea* (Figure 4b). To the best of our knowledge, this is both the first detailed description of the tocochromanol profile and the first report of αT1 in *M. subterranea*. 11′‐αT1 was the major αT1 isomer in *M. subterranea*, in agreement with previous results in cultured marine and freshwater microalgae from genera *Nannochloropsis*, *Chlorella*, and *Tetraselmis* (Montoya‐Arroyo, Lehnert, Lux, et al., 2022; Montoya‐Arroyo, Lehnert, Muñoz‐González, et al., 2022), but in contrast with existing reports in marine phytoplankton, where only the 12′‐αT1 isomer has been reported so far (Dunlap et al., 2002; Yamamoto et al., 2001).

**FIGURE 5:**
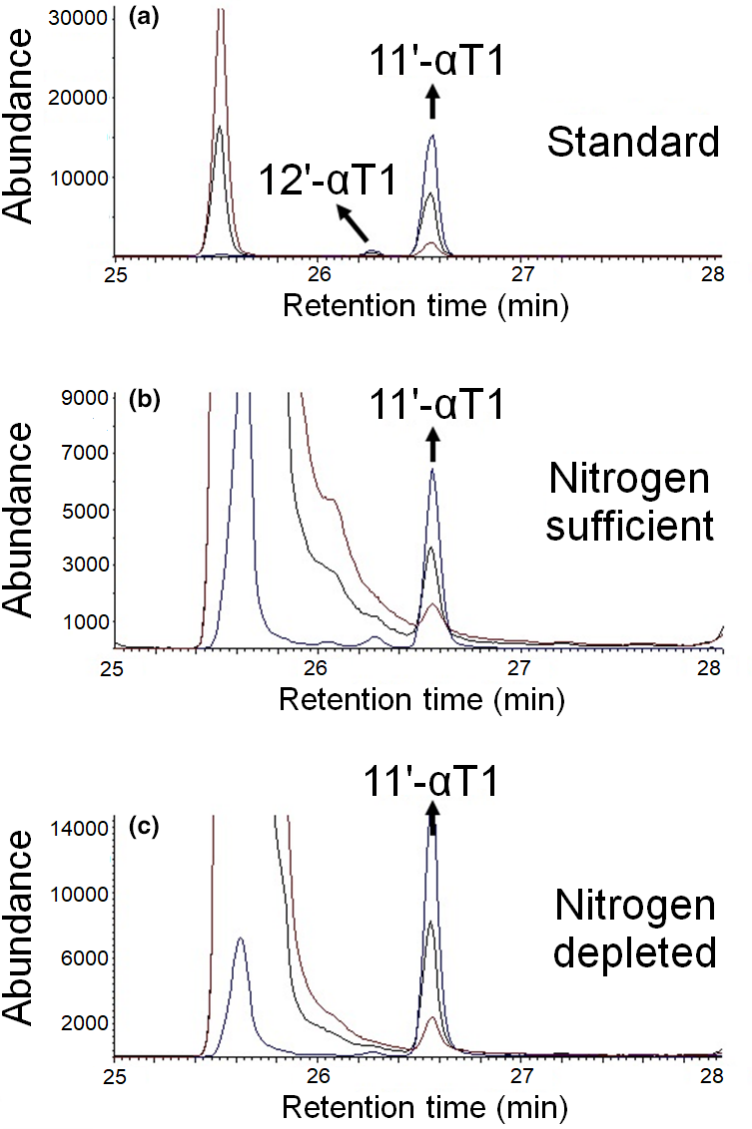
Identification of 11′‐α‐tocomonoenol (11′‐αT1) in *Monodopsis subterranea* cultured using GC–MS: Representative GC–MS chromatograms of isolated 11′‐α‐tocomonoenol (11′‐αT1) standard containing traces of 12′‐α‐tocomonoenol (12′‐αT1) (a), and *M. subterranea* biomass under nitrogen‐sufficient (b), and nitrogen‐depleted conditions (c). Red line: ion *m*/*z* 502.5 (molecular ion, αT); blue line: ion *m*/*z* 500.2 (molecular ion, αT1); black line: ion *m*/*z* 237.2 (tropylium ion, α‐congener).

Figure 5 shows the corresponding GC–MS chromatogram of *M. subterranea* extracts compared to one of isolated 11′‐αT1 (Montoya‐Arroyo, Lehnert, Lux, et al., 2022; Müller et al., 2018). The intensity of the molecular ion (*m*/*z* 500) of 12′‐αT1 represented just 3.8 ± 3.9% and 7.3 ± 4.6% of the total intensity observed for 11′‐αT1 under nitrogen‐sufficient and nitrogen‐depleted conditions, respectively. No significant differences in the concentrations of 12′‐αT1 were observed between the two culture conditions (*p* = .378).

Two potential mechanisms could explain the accumulation of αT in the absence of accumulation of T1 or T3 isomers during nitrogen depletion: (i) an increased biosynthesis of T, but not of that of T1 and T3, and (ii) a reduced oxidation of T compared to T1 and T3. Nitrogen depletion under phototropic conditions might favor the biosynthesis of T and not that of T1 and T3, since phototrophic conditions might provide the NADPH required for the reduction of geranyl‐geranyl‐pyrophosphate to phytyl‐pyrophosphate (Keller et al., 1998), limiting the availability of unsaturated and partially saturated sidechain‐precursors required for the biosynthesis of T3 and T1, respectively (Pellaud et al., 2018).

In addition, the breakdown of chlorophyll generates phytol that can be phosphorylated to provide saturated phytyl‐pyrophosphate for T biosynthesis (Valentin et al., 2006), but not for the synthesis of T3 and T1 (Pellaud et al., 2018). Phytol‐recycling from chlorophyll has been shown to be a critical determinant of T biosynthesis (vom Dorp et al., 2015) and might be consistent with the reduced chlorophyll concentrations observed in nitrogen‐depleted biomass in the present experiment (Figure 2). T1 might also be synthesized from the mono‐unsaturated sidechains derived from the breakdown of chlorophyll precursors (Albert et al., 2022); however, the availability of such mono‐unsaturated chlorophyll precursors might be limited due to the sufficient light exposure of our *M. subterranea* in the present experiment.

However, the relatively stable absolute contents of αT1 and T3 under both culture conditions suggest that the biosynthesis of T1 and T3 might occur independently of nitrogen availability and independently from the synthesis of αT. The 11′‐αT1 isomer, which was the major αT1 in *M. subterranea* and other microalgae (Montoya‐Arroyo, Lehnert, Lux, et al., 2022; Montoya‐Arroyo, Lehnert, Muñoz‐González, et al., 2022), has its single double bond of the sidechain in a position consistent with that of those isoprenoids derived from the non‐mevalonate pathway (Pellaud et al., 2018), the pathway used for biosynthesis of tocochromanol sidechains in microalgae and cyanobacteria (Kato & Hasunuma, 2021; Schwender et al., 1997) and land plants (Schwender et al., 1997). It has been suggested that 12′‐αT1 might originate from a different biosynthetic precursor (Pellaud et al., 2018) or as a consequence of enzymatic modification of the sidechain of already synthetized tocochromanols (Fujisawa et al., 2010), but further evidence is required to confirm the biosynthetic origin of this isomer.

In microalgae, nitrogen depletion promotes oxidative stress (Zhang et al., 2013), decreases photosynthetic efficiency, and increases light sensitivity (Zhao et al., 2017), which all together might increase tocochromanol oxidation. Hence, the increase in αT and stable concentrations of T1 and T3 in nitrogen‐depleted microalgae might also be explained by higher oxidation of T1 and T3, which have higher intramembrane mobility compared to αT (Serbinova et al., 1991; Yamamoto et al., 2001), and in consequence might oxidize faster under the pro‐oxidant conditions (Yoshida et al., 2003) under nitrogen depletion. Furthermore, it has been reported that the unsaturated fatty acids eicosapentaenoic acid (EPA) and oleic acid may even increase in *M. subterranea* grown in a similar experiment under nitrogen depletion (Frick et al., 2023), which can also favor higher oxidation of tocochromanols in microalgae under this specific culture condition.

Light availability reduces T1 in the leaves of land plants (Kruk et al., 2011; Threlfall & Whistance, 1977), probably due to its role on NADPH availability and activity of reductase enzymes (Pellaud et al., 2018), but, to the best of our knowledge, no information exists regarding the effect of light on T1 content in aquatic photosynthetic organisms or on the effects of nitrogen depletion on T1 in land plants. In general, limited knowledge exists on factors affecting T1 concentrations in land plants and the present work is the first to address these factors in aquatic photosynthetic organisms. Further experiments on microalgal and plant physiology investigating the effects of abiotic and biotic factors involved in vitamin E biosynthesis are warranted in order to provide additional information on mechanistic determinants of biosynthesis and the accumulation of T1 in photosynthetic organisms. The present data indicates that T1 biosynthesis and accumulation in photosynthetic organisms might be similar to those in land plants, which is in agreement with shared biosynthetic pathways (Kato & Hasunuma, 2021; Schwender et al., 1997).

Both the absolute (7.64 ± 1.90 mg/kg DW) and relative contents of αT1 in *M. subterranea* in nitrogen‐sufficient conditions were higher compared to the Eustigmatophyceae microalgae *Nannochloropsis oceanica* (2.46 ± 0.71 mg/kg DW and 0.6% of tocochromanols) (Montoya‐Arroyo, Lehnert, Lux, et al., 2022) and *Nannochloropsis limnetica* (traces) (Montoya‐Arroyo, Lehnert, Muñoz‐González, et al., 2022). In comparison to microalgae from Chlorophyceae, *M. subterranea* had higher absolute and relative αT1 contents compared to *Chlorella sorokiniana* (0.82 ± 1.43 mg/kg and 0.10 ± 0.20% of total tocochromanol content) and *Tetraselmis suecica* (5.44 ± 4.83 mg/kg and 0.89 ± 0.80% of tocochromanols) (Montoya‐Arroyo, Lehnert, Muñoz‐González, et al., 2022), but did not reach the reported content for *Tetraselmis* sp. (15.20 ± 3.03 mg/kg DW and 17% of tocochromanols) (Montoya‐Arroyo, Lehnert, Lux, et al., 2022). αT1 contents in vegetable oils range from 0 to around 5% of total tocochromanols (Irías‐Mata et al., 2017; Ng et al., 2004; Puah et al., 2007; Rammell & Hoogenboom, 1985), and in fish and fish‐derived products, they reach up to 20% (Dunlap et al., 2002; Gotoh et al., 2011; Yamamoto et al., 2001).

The presence of T3 has been scarcely reported in microalgae, but previous results from us (Montoya‐Arroyo, Lehnert, Lux, et al., 2022; Montoya‐Arroyo, Lehnert, Muñoz‐González, et al., 2022) and others (Ruggeri et al., 1985; Yusof et al., 2011) indicate the presence of minor quantities of T3 in microalgae from different species. In *M. subterranea*, the content of αT1 exceeds by far the content of T3. For both absolute and relative values, αT1 represents ca. 5 times the observed content of total T3.

Similar results have been reported for *Nannochloropsis oceanica*, *Tetraselmis* sp., *Chlorella vulgaris* (Montoya‐Arroyo, Lehnert, Lux, et al., 2022), *Nannochloropsis limnetica*, and *Tetraselsmis suecica* (Montoya‐Arroyo, Lehnert, Muñoz‐González, et al., 2022), in which the content of αT1 tended to be higher than that of total T3. Particularly, the αT1 content in *Tetraselmis* sp. exceeded that of T3 by 20 times (Montoya‐Arroyo, Lehnert, Lux, et al., 2022). Of the microalgae containing both αT1 and T3 (Montoya‐Arroyo, Lehnert, Lux, et al., 2022; Montoya‐Arroyo, Lehnert, Muñoz‐González, et al., 2022), only *Chlorella sorokiniana* has been reported to contain a higher content of T3 than αT1 (Montoya‐Arroyo, Lehnert, Muñoz‐González, et al., 2022). This is in agreement with data from pumpkin seed oil, where T1 content is higher than the major T3 (Butinar et al., 2011), but clearly contrasts with data from palm oil, where αT1 represents only a minor compound in comparison to T3 (Irías‐Mata et al., 2017).

Together with previous reports, the data of the present work indicates that microalgae are a source of αT1, particularly 11′‐αT1 and, to a lower extent, 12′‐αT1. Nitrogen depletion significantly increased the content of total tocochromanols and αT, as previously hypothesized, but did not significantly alter the accumulation of tocochromanols with unsaturated sidechains, such as T1 and T3. This indicates that the accumulation of T1 and T3 in microalgae is independent of that of αT, as it occurs in land plants.

## 4 CONCLUSION

αT is the quantitatively predominant tocochromanol in *M. subterranea*. αT1 was detected mostly in the form of 11′‐αT1, and its content was five times higher than that of the sum of all T3. Nitrogen depletion of the microalga increased tocopherols, but not αT1 or T3. Nitrogen depletion during culture may thus be a promising strategy to increase the vitamin E content of microalgae in general and *M. subterranea* in particular, but it does not affect αT1 content, indicating that αT1 accumulation in these microalgae is independent of that of αT.

## AUTHOR CONTRIBUTIONS

**Alexander Montoya‐Arroyo:** Conceptualization (lead); data curation (lead); formal analysis (lead); investigation (lead); methodology (lead); project administration (lead); writing – original draft (lead); writing – review and editing (lead). **Alejandra Muñoz‐González:** Formal analysis (supporting); investigation (supporting); methodology (equal); writing – review and editing (supporting). **Katja Lehnert:** Data curation (supporting); formal analysis (supporting); investigation (supporting); methodology (supporting); validation (equal); writing – review and editing (supporting). **Konstantin Frick:** Investigation (supporting); methodology (supporting); project administration (supporting); writing – review and editing (supporting). **Ulrike Schmid‐Staiger:** Conceptualization (supporting); project administration (supporting); supervision (supporting); writing – review and editing (supporting). **Walter Vetter:** Conceptualization (supporting); formal analysis (supporting); project administration (supporting); supervision (supporting); writing – review and editing (supporting). **Jan Frank:** Conceptualization (lead); formal analysis (supporting); project administration (supporting); supervision (lead); writing – original draft (lead); writing – review and editing (lead).

## CONFLICT OF INTEREST STATEMENT

The authors declare that there are no conflicts of interest.

## Supporting information


Appendix S1.

## Data Availability

The data that support the findings of this study are available from the corresponding author upon reasonable request.
