# Supplementary material for: Monodopsis subterranea is a source of α‐tocomonoenol, and its concentration, in contrast to α‐tocopherol, is not affected by nitrogen depletion
Source: Food Sci Nutr. 2023 Dec 20;12(3):1869–79. doi: 10.1002/fsn3.3880 (PMC10916641; doi:10.1002/fsn3.3880)
Supplement: Supplementary file 1 — Appendix S1. [file FSN3-12-1869-s001.pdf]

Monodopsis subterranea is a source of α-tocomonoenol and its concentration, in contrast to α-tocopherol, is not affected by nitrogen depletion

Alexander Montoya-Arroyo [1], Alejandra Muñoz-González [1,2], Katja Lehnert [3], Konstantin Frick [4], Ulrike Schmid-Staiger [5], Walter Vetter [3], Jan Frank [1]

1) Institute of Nutritional Sciences (140b), University of Hohenheim, Garbenstr. 28, 70599 Stuttgart, Germany
2) School of Food Technology, University of Costa Rica, 2060 San Pedro, Costa Rica
3) Institute of Food Chemistry (170b), University of Hohenheim, 70599 Stuttgart, Germany
4) Institute of Interfacial Process Engineering and Plasma Technology, University of Stuttgart, 70569 Stuttgart, Germany
5) Innovation Field Functional Ingredients, Fraunhofer Institute for Interfacial Engineering and Biotechnology IGB, 70569 Stuttgart

(*) Corresponding author: Alexander Montoya-Arroyo, University of Hohenheim, Institute of Nutritional Sciences, Department of Food Biofunctionality (140b), Garbenstr. 28, 70599 Stuttgart, Germany; alexander.montoya@nutres.de

Supplementary Material

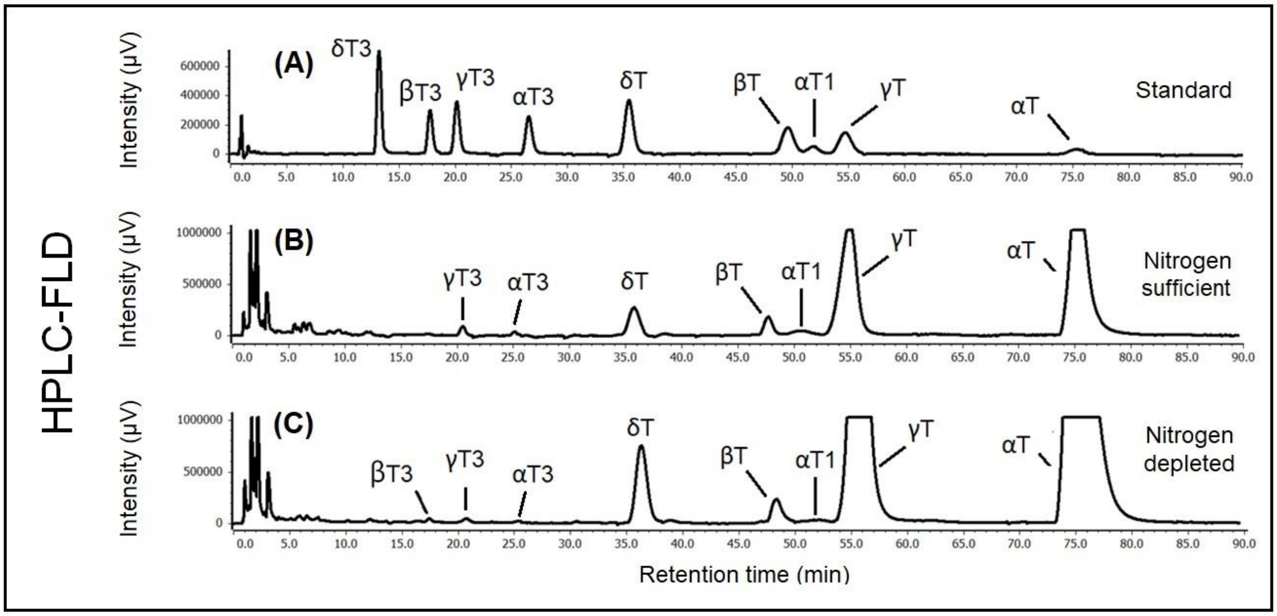

Figure S1. Representative HPLC-FLD chromatograms of tocochromanol standards including tocopherols (T); 11'-α-tocomonoenol (αT1), and tocotrienols (T3) **(A)**; and tocochromanol profile of *Monodopsis subterranea* biomass under nitrogen-sufficient **(B)** and nitrogen-depleted conditions **(C)**.

Table S1. Fragmentation patterns for targeted analysis of αT1 using LC-APCI-MS^n in nitrogen-sufficient and nitrogen-depleted *Monodopsis subterranea*.

Sample	RT (min)	Identified Ions	Detection
11'-α-tocomonoenol standard	12.73	*m/z* 429.3713 ($C_{29}H_{49}O_2$; Δ ppm = -3.2) ($[M+H]^+$)<br>*m/z* 205.1220 ($C_{13}H_{17}O_2$; Δ ppm = -1.5)<br>*m/z* 165.0909 ($C_{10}H_{13}O_2$; Δ ppm = -0.8)<br>*m/z* 69.0706 (C_5H_9; Δ ppm = +10.6)	MS^2
Nitrogen-sufficient	12.75	*m/z* 429.3717 ($C_{29}H_{49}O_2$; Δ ppm = -4.3) ($[M+H]^{+)}$<br>*m/z* 205.1221 ($C_{13}H_{17}O_2$; Δ ppm = -3.7)<br>*m/z* 165.0907($C_{10}H_{13}O_2$; Δ ppm = -5.2)<br>*m/z* 69.0704 (C_5H_9; Δ ppm = -0.8)	MS^2
Nitrogen-depleted	12.80	*m/z* 429.3714 ($C_{29}H_{49}O_2$; Δ ppm = -2.5) ($[M+H]^+$)<br>*m/z* 205.1222 ($C_{13}H_{17}O_2$; Δ ppm = -0.4)<br>*m/z* 165.0908 ($C_{10}H_{13}O_2$; Δ ppm = -1.3)<br>*m/z* 69.0705 (C_5H_9; Δ ppm = +9.5)	MS^2

Table S2. Fragmentation patterns for tocotrienols in *Monodopsis subterranea* under nitrogen-sufficient and nitrogen-depleted conditions using LC-APCI-MS.

Sample	Congener	RT (min)	Identified ions	Detection
Standard	δ-tocotrienol	8.24	*m/z* 397.3092 ($C_{27}H_{41}O_2$; Δ ppm = -2.2) ($[M+H]^+$)<br>*m/z* 177.0907 ($C_{11}H_{13}O_2$; Δ ppm = -1.6)<br>*m/z* 137.0595 ($C_8H_9O_2$; Δ ppm = -1.2)<br>*m/z* 69.0706 (C_5H_9; Δ ppm = +11.1)	MS^2
	β-tocotrienol	9.27	*m/z* 411.3247 ($C_{28}H_{43}O_2$; Δ ppm = -2.6) ($[M+H]^+$)<br>*m/z* 191.1063 ($C_{12}H_{15}O_2$; Δ ppm = -1.8)<br>*m/z* 151.0751 ($C_9H_{11}O_2$; Δ ppm = -1.5)<br>*m/z* 69.0705 (C_5H_9; Δ ppm = +9.6)	MS^2
	γ-tocotrienol	9.83	*m/z* 411.3245 ($C_{28}H_{43}O_2$; Δ ppm = -3.0) ($[M+H]^+$)<br>*m/z* 191.1063 ($C_{12}H_{15}O_2$; Δ ppm = -2.1)<br>*m/z* 151.0752 ($C_9H_{11}O_2$; Δ ppm = -1.4)<br>*m/z* 69.0706 (C_5H_9; Δ ppm = +10.2)	MS^2
	α-tocotrienol	10.71	*m/z* 425.3398 ($C_{29}H_{45}O_2$; Δ ppm = -3.6) ($[M+H]^+$)<br>*m/z* 205.1222 ($C_{13}H_{17}O_2$; Δ ppm = -0.6)<br>*m/z* 165.0908 ($C_{10}H_{13}O_2$; Δ ppm = -1.2)<br>*m/z* 69.0705 (C_5H_9; Δ ppm = +8.6)	MS^2
Nitrogen- sufficient	δ-tocotrienol	8.23	*m/z* 397.3459 ($C_{27}H_{41}O_2$; Δ ppm = -3.0) ($[M+H]^+$)<br>*m/z* 177.0909 ($C_{11}H_{13}O_2$; Δ ppm = -3.9)<br>*m/z* 137.0596 ($C_8H_9O_2$; Δ ppm = -4.9)	MS
	β-tocotrienol	9.24	*m/z* 411.3615 ($C_{28}H_{43}O_2$; Δ ppm = -2.8) ($[M+H]^+$)<br>*m/z* 191.1066 ($C_{12}H_{15}O_2$; Δ ppm = -3.0)<br>*m/z* 151.0753 ($C_9H_{11}O_2$; Δ ppm = -4.0)	MS
	γ-tocotrienol	9.80	*m/z* 411.3246 ($C_{28}H_{43}O_2$; Δ ppm = -4.0) ($[M+H]^+$)<br>*m/z* 191.1065 ($C_{12}H_{15}O_2$; Δ ppm = -3.5)<br>*m/z* 151.0753 ($C_9H_{11}O_2$; Δ ppm = -4.2)	MS

	α-tocotrienol	10.71	*m/z* 425.3398 ($C_{29}H_{45}O_2$; Δ ppm = -5.0) ($[M+H]^+$)<br>*m/z* 205.1222 ($C_{13}H_{17}O_2$; Δ ppm = -3.3)<br>*m/z* 165.0910 ($C_{10}H_{13}O_2$; Δ ppm = -3.6)	MS
Nitrogen-depleted	δ-tocotrienol	8.19	*m/z* 397.3096 ($C_{27}H_{41}O_2$; Δ ppm = -2.6) ($[M+H]^+$)<br>*m/z* 177.0909 ($C_{11}H_{13}O_2$; Δ ppm = -3.8)<br>*m/z* 137.0596 ($C_8H_9O_2$; Δ ppm = -4.8)	MS
	β-tocotrienol	9.35	*m/z* 411.3243 ($C_{28}H_{43}O_2$; Δ ppm = -4.9) ($[M+H]^+$)<br>*m/z* 191.1064 ($C_{12}H_{15}O_2$; Δ ppm = -4.5)<br>*m/z* 151.0751 ($C_9H_{11}O_2$; Δ ppm = -5.1)	MS
	γ-tocotrienol	9.85	*m/z* 411.3255 ($C_{28}H_{43}O_2$; Δ ppm = -1.9) ($[M+H]^+$)<br>*m/z* 191.1065 ($C_{12}H_{15}O_2$; Δ ppm = -3.6)<br>*m/z* 151.0752 ($C_9H_{11}O_2$; Δ ppm = -4.6)	MS
	α-tocotrienol	10.76	*m/z* 425.3398 ($C_{29}H_{45}O_2$; Δ ppm = -5.1) ($[M+H]^+$)<br>*m/z* 205.1222 ($C_{13}H_{17}O_2$; Δ ppm = -3.1)<br>*m/z* 165.0910 ($C_{10}H_{13}O_2$; Δ ppm = -3.6)	MS
